# Precancerous Stem Cells Can Serve As Tumor Vasculogenic Progenitors

**DOI:** 10.1371/journal.pone.0001652

**Published:** 2008-02-20

**Authors:** Rulong Shen, Yin Ye, Li Chen, Qingtao Yan, Sanford H. Barsky, Jian-Xin Gao

**Affiliations:** Department of Pathology and Comprehensive Cancer Center, Ohio State University, Columbus, Ohio, United States of America; Baylor College of Medicine, United States of America

## Abstract

Tumor neo-vascularization is critical for tumor growth, invasion and metastasis, which has been considered to be mediated by a mechanism of angiogenesis. However, histopathological studies have suggested that tumor cells might be the progenitor for tumor vasculature. Recently, we have reported that the precancerous stem cells (pCSCs) representing the early stage of developing cancer stem cells (CSCs), have the potential for both benign and malignant differentiation. Therefore, we investigated whether pCSCs serve as progenitors for tumor vasculogenesis. Herein, we report that in the pCSC-derived tumors, most blood vessels were derived from pCSCs. Some pCSCs constitutively expressed vasculogenic receptor VEGFR-2, which can be up-regulated by hypoxia and angiogenesis-promoting cytokines, such as GM-CSF, Flt3 ligand, and IL-13. The pCSCs are much more potent in tumor vasculogenesis than the differentiated tumor monocytic cells (TMCs) from the same tumor, which had comparable or even higher capacity to produce some vascular growth factors, suggesting that the potent tumor vasculogenesis of pCSCs is associated with their intrinsic stem-like property. Consistently tumor vasculogenesis was also observed in human cancers such as cervical cancer and breast cancer and xenograft lymphoma. Our studies indicate that pCSCs can serve as tumor vasculogenic stem/progenitor cells (TVPCs), and may explain why anti-angiogenic cancer therapy trials are facing challenge.

## Introduction

Tumor malignancy is determined by the growth rate, invasiveness and metastasis of tumor. The growth rate, invasiveness and outcome of metastasis are in turn dependent on the establishment of tumor vascular network, which provides nutrients required for cancer cell expansion and drains away wastes produced by cancer cells [1]–[5]. The prevailing concept holds that the tumor vasculature is formed via a mechanism of tumor angiogenesis: pre-existing blood vessels, which are stimulated by various angiogenic growth factors secreted by cancer cells, such as vascular endothelial growth factor (VEGF), angiopoietin (Ang)-1, and Ang-2, sprout into cancer cell clusters to form tumor vasculature [1], [2]. VEGF binds receptors VEGFR-1 (Flt1) and VEGFR-2 (KDR/Flk1); and the latter appears to mediate almost all of the known cellular response to VEGF [6]–[8]. While the existence of tumor vasculogenesis remains controversial [4], [9]–[14], recent studies on human melanoma and brain tumors have suggested that most tumor vasculature might be derived from cancer cells rather than preexisting vessels [12]–[14], implicating a mechanism of tumor vasculogenesis, which might play an important role in tumorigenesis.

Vasculogenesis is a process of blood vessel formation through *de novo* production of endothelial cells, originally observed during the embryonic development. Tumor vasculogenesis denotes that some cancer cells can spontaneously form blood vessels in tumor via transdifferentiation into endothelial-like cells. A cancer resembles a neo-organ, composed of various stages of developing cancer cells, including precancerous stem cells (pCSCs) [15]–[17], cancer stem cells (CSCs) [18]–[21] and cancer cells [16]. A cancer cell is supposed to be developed from a process of tumor-initiating cells (TICs)→pCSCs→CSCs→cancer cells [16]. CSCs have been shown to promote tumor angiogenesis through secreting growth factor VEGF [22], although their potential for transdifferentiation into endothelial cells has not been demonstrated. Recently, we have discovered that the precancerous stem cells (pCSCs), representing the premalignant stage of developing CSCs, have the potential for both benign and malignant differentiation [15], [16]. The pCSCs can develop into tumors in severe combined immunodeficiency disease (SCID) mice, but not in bone marrow (BM)-reconstituted (BMR) mice, blastocyst chimera mice, and immunocompetent (IC) mice [15]. In the BMR mice, however, pCSCs can differentiate into various types of tissue cells, including endothelial-like cells, suggesting that they might have the capacity to form blood vessels in tumorigenic environments [15]. To verify the hypothesis, we have investigated the tumor vasculogenic capacity of pCSCs in a murine model of lymphoma [15], [23]. In the pCSC-derived tumor, tumor blood vessels were essentially derived from transplanted pCSCs. The pCSCs are much more potent in tumor neo-vascularization compared to the differentiated tumor monocytic cells (TMCs) derived from the same tumor. Various types of human cancer cell lines that should contain pCSCs and CSCs [24] also exhibited the potential for tumor vasculogenesis in tumor xenografts. Tumor vascular endothelial cells were inevitably defective in phenotype and function with remarkable variations between individuals. Consistently, most endothelial cells were abnormal in human tumor vasculature. Thus, pCSCs can serve as tumor vasculogenic stem/progenitor cells. The finding may explain why anti-angiogenic cancer therapy trails are facing serious challenge [25].

## Results

### Precancerous stem cells serve as progenitors for tumor vasculogenesis

**Figure 1:**
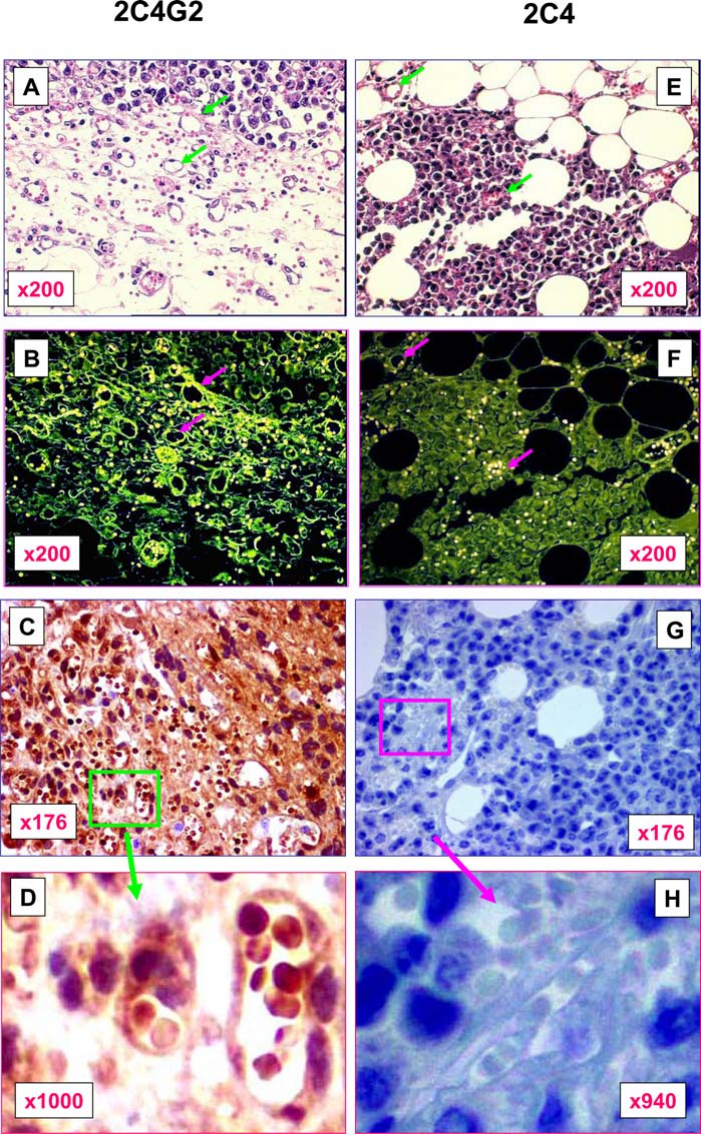
The contribution of pCSCs to tumor vasculogenesis. The pCSCs (clone 2C4 or 2C4G2) were inoculated s.c. or i.p. (5×10^6^/mouse; n = 10/group) into SCID mice. About 40∼100% of the mice developed tumors, which grew so fast once they were palpable that the mice had to be sacrificed within 7 d of the palpation [15]. The tumors were harvested and fixed with 10% formalin in PBS. The sections were stained with H & E, and subjected to microscopic analysis simultaneously under the bright (A & E) and fluorescent fields (B & F), respectively (A & B). To verify the results from fluorescent microscopy, successive sections were subjected to IHC staining with rabbit mAb to GFP (1∶300 dilution) followed by HRP-conjugated goat anti-rabbit IgG (C, D, G & H). The data shown are a representative of tumor micrographs. A–D, A tumor from a mouse inoculated with GFP-expressing cells (2C4G2); the insets in C were enlarged as shown in D, demonstrating GFP-positive TVECs and RBCs. E–H, A tumor from a mouse transplanted with non-GFP-expressing cells (2C4), and the insets in G were enlarged as shown in H, demonstrating GFP-negative TVECs and RBCs. C and G are successive sections of A and E, respectively, and were stained with the same rabbit mAb to GFP. Arrows in A and E indicate the same blood vessels in B and F, respectively, which were GFP-positive or GFP-negative.

**Figure 2:**
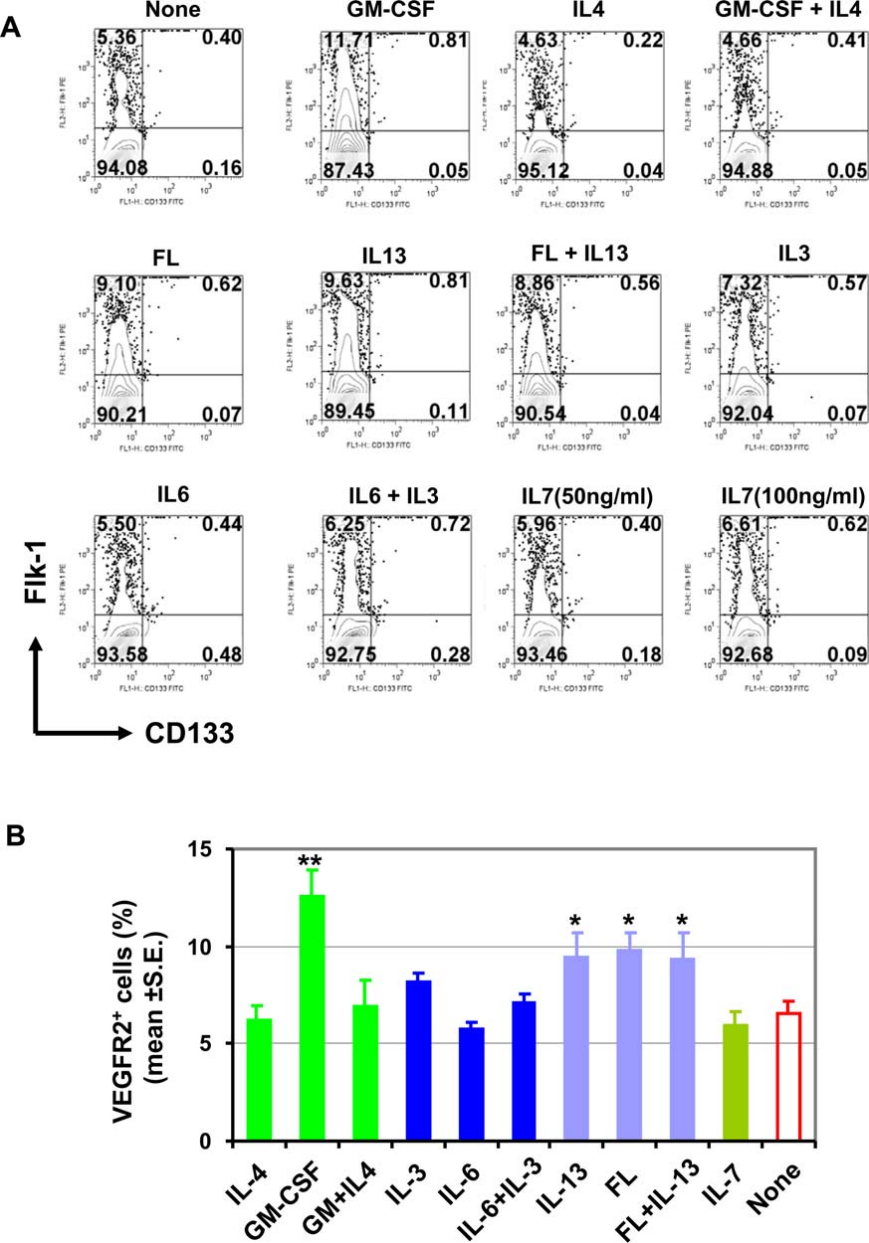
The effect of cytokines on VEGFR-2 expression in pCSCs. The pCSCs (2C4 clone) were cultured in 2.0 ml of R10F (1×10^5 ^cells/well) in 24-well plates in the presence of cytokine IL-3 (50 ng/ml), IL-4 (20 ng/ml), IL-6 (50 ng/ml); IL-7 (50 or 100 ng/ml), IL-13 (50 ng/ml), GM-CSF (40 ng/ml), or FL (200 ng/ml) alone or in combination (GM-CSF+IL-4; FL+IL-13; IL-3+IL-6). Control cultures were absent from exogenous cytokines. The cells were harvested 3 days later, stained with FITC-conjugated rat mAb to murine CD133 and PE-conjugated mAb to murine Flk-1 (VEGFR-2), and analyzed by flow cytometry [15]. A, Data shown are contour plots of a representative experiment. The numbers in quadrants indicate the percentage of each subpopulation. B, Shown is the percentage of Flk-1^+^ cells of pCSCs derived from four independent experiments. **, p<0.01; * p<0.05, compared to the cultures without exogenous cytokines.

Since pCSCs has the potential to differentiate into endothelial-like cells [15], we hypothesized that pCSCs may mediate tumor vasculogenesis. To test the hypothesis, we transplanted intraperitoneally (i.p.) or subcutaneously (s.c.) pCSCs (clone 2C4) or enhanced green fluorescent protein (GFP)-expressing pCSCs (clone 2C4G2 derived from 2C4 clone) into SCID mice, as previously described [15]. The tumors were removed at the size ∼10 mm in diameter, and prepared for paraffin-embedded sections. The sections were stained with H & E and subjected to light and fluorescent microscopic analysis. This method allowed us to precisely identify and photograph pCSC-derived GFP^+^ endothelial-like cells in well-preserved tumor vasculature. As shown in Figure 1, mature blood vessels were observed in both 2C4G2 (Fig. 1A) and 2C4 (Fig. 1E) cell-derived tumors. In some areas, the pCSC-derived cells were coalesced into the primary capillary plexus, developing into massive capillary beds within tumors (Fig. 1).

All tumor cells derived from 2C4G2 clone exhibited strong GFP-specific green fluorescence (Fig. 1B); whereas the tumor cells derived from 2C4 clone demonstrated weak, non-specific auto-fluorescence (Fig. 1F). Importantly almost all tumor blood vessels or capillaries exhibited strong GFP-specific green fluorescence in the 2C4G2-derived, but not in 2C4-derived tumors, regardless of i.p. or s.c. transplanted tumors (Fig. 1B and F; red arrows indicate representative vessels, which are the same as in Fig. 1A and E indicated by green arrows), suggesting that not only tumor cells but also tumor vascular endothelial cells (TVECs) can be derived from pCSCs.

Since paraffin-embedded tissue sections usually demonstrate high background of auto-fluorescence, we further verified pCSC-derived TVECs in tumors by immunohistochemical (IHC) staining of successive sections (Fig. 1C & G). Successive sections from the same tumors either derived from 2C4G2 or 2C4 cells were stained with the same high affinity rabbit monoclonal (mAb) antibody to GFP followed by secondary HRP-conjugated goat anti-rabbit IgG. As expected, the sections of 2C4G2 (Fig. 1C), but not 2C4 (Fig. 1G) cell-derived tumors demonstrated GFP-positive tumor cells as well as GFP-positive blood vessels, consistently with the observations under fluorescent microscope (Fig. 1B & F). Endothelial cells of capillaries in 2C4G2 cell-derived tumors were strongly stained in nuclei and cytoplasm by mAb to GFP (Fig. 1D); in contrast, no endothelial cells were stained by the same mAb to GFP in 2C4 cell-derived tumors (Fig. 1H). Moreover, pCSCs not only serve as progenitors for capillary endothelial cells, but also for the endothelial cells of larger blood vessels, because endothelial cells in various sizes of blood vessel of the tumors were GFP-positive (Fig. 1 & Supplementary (s) Fig. 1C,E,F). Endothelial cells were stained either in cytoplasm alone (Fig. S1E) or in both cytoplasm and nuclei (Fig. S1F). Some of them may be weakly stained (Fig. S1H). The weak stained endothelial cells were unlikely non-specific, because the same antibody did stain endothelial cells in 2C4 cell-derived tumors (Fig. 1G, H, & Fig. S1A, B). This is consistent with our previous report that GFP in pCSCs can be down-regulated when they were differentiating into lineage-specific progenies, as demonstrated by flow cytometry [15]. The finding supports that GFP may be down-regulated or inactivated when progenitors differentiate into progenies [26].

The high affinity of rabbit mAb to GFP greatly promoted specificity of IHC staining. The high specificity lends us capability to directly investigate multipotency of pCSCs in well-preserved tissue sections with almost null background (Fig. 1G). With the advantage, we also identified pCSC-derived red blood cells (RBCs) in tumors (Fig. 1D). In the capillary beds of 2C4G2 cell-derived tumors, many of RBCs were GFP-positive with variable levels (Fig. 1C & D). Even in the bigger blood vessels, GFP-positive RBCs were also observed together with GPF-negative RBCs (Fig. S1F,G). These GFP-positive RBCs are truly derived from 2C4G2 pCSCs, because no GFP-positive RBCs were detected in the 2C4 tumors using the same mAb to GFP (Fig. 1G & H, and Fig. S1A, B). The significance of pCSC-derived erythrocytes in tumors needs further investigation.

Taken together, the results directly confirm our previous finding that pCSCs have the multipotency of differentiation [5], [15]. Especially, the pCSCs can serve as tumor vasculogenic stem/progenitor cells (TVPCs) to differentiate into TVECs and RBCs, resembling hemangioblasts [27].

### Precancerous stem cells express vascular progenitor marker VEGFR-2

Both angiogenesis and vasculogenesis are mediated by VEGF through binding VEGFR [28]. Receptor 2 for VEGF (VEGFR-2) is most important for angiogenesis and vasculogenesis, because it mediates almost all cellular response to VEGF [28]. Moreover, VEGFR-2 is not only expressed on endothelial cells to promote their proliferation [29], but also on the stem/progenitor cells to mediate vasculogenesis [8]. To investigate whether the vasculogenic capacity of pCSCs is associated with VEFGR2-mediated signaling pathway, we first examined whether pCSCs constitutively expressed VEGFR-2 by flow cytometry. As shown in Figure 2, ∼5% of 2C4 cells constitutively expressed VEGFR-2 when cultured *in vitro* without exogenous cytokines, although they did not express CD133, a marker for normal endothelial progenitors [30]. To determine whether VEGFR-2 expression on pCSCs is regulated by tumor environmental cues, we examined the effects of cytokines on the expression *in vitro*., including Flt3 ligand (FL) [31], GM-CSF [32], IL-3 [33], IL-4 [34], IL-6 [35], IL-7 [36] and IL-13 [37]. These cytokines can be detected in tumor environments and have been reported having effects on tumor angiogenesis. 2C4 cells were cultured for 3 days in the presence of a cytokine alone or in combination, and analyzed for cell proliferation and VEGFR-2 expression (Fig. 2). While all the cytokines appeared to promote pCSC expansion except for IL-6, the viable cell counts were marginally or significantly increased only in the cultures with GM-CSF, IL-13, FL, or IL-13 plus FL, compared to the cultures absent of exogenous cytokines (Fig. S2). There was no significant synergistic or additive effect between them (Fig. S2). VEGFR-2 expression on pCSCs was significantly up-regulated by FL, IL-13, and GM-CSF, but not by IL-3, IL-4, IL-6 and IL-7 (Fig. 2). Interestingly, IL-4 almost completely inhibited VEGFR-2 expression induced by GM-CSF (Fig. 2). This may explain why IL-4 has strong anti-tumor activity [34], [38]. Although VEGFR-2 expression on pCSCs was significantly up-regulated by FL, IL-13 and GM-CSF, only few VEGFR-2^+^ cells expressed CD133 (Fig. 2A), indicating that VEGFR-2-expressing pCSCs is essentially distinct from normal endothelial progenitors [30]. The result suggests that the vasculogenic capacity of pCSCs is associated with VEGFR-2 expression.

### Precancerous stem cells can differentiate into endothelial-like cells in responding to hypoxia

**Figure 3:**
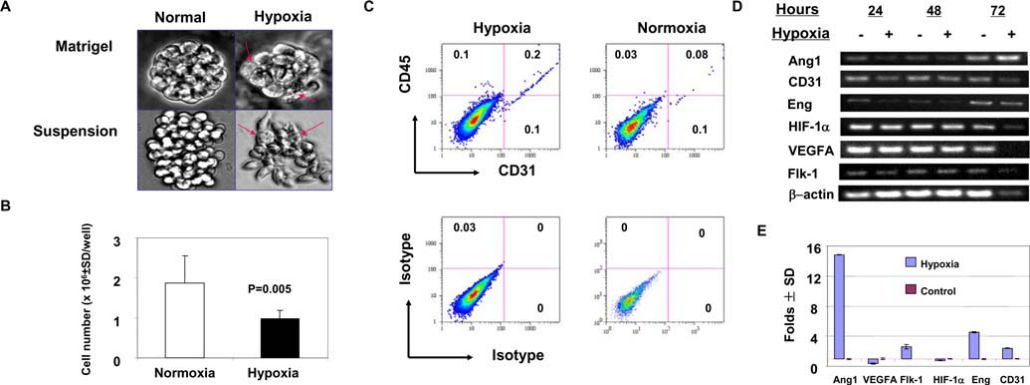
Differentiation of pCSCs in responding to hypoxia. The pCSCs (clone 2C4; 5×10^4^/well) were cultured in 0.5 ml (hypoxia) or 2.0 ml (normoxic) of R10F medium or Matrigel. From day 3∼4 of cultures, the cells in the suspension medium or Matrigel are morphologically altered in the hypoxic culture but not in normoxic cultures (A). The cells were harvested on day 4 of cultures, enumerated (B), and analyzed for CD31 and CD45 expression by flow cytometry (C), or harvested on day 1, 2, 3 and 4 for angiogenic factor expression revealed by RT-PCR (D, day 1∼3) or real-time PCR (E, day 4). A, the phase contrast microphotographs of cell morphology of pCSCs cultured in Matrigel-containing or suspension medium under the normal or hypoxic condition, which were taken at day 4 of culture. Arrows indicate apoptotic cells. B, Hypoxia inhibited proliferation of pCSCs. Data shown are pooled results from two of four reproducible experiments (n = 4 well/group/expt). C, phenotypic analysis of the pCSCs cultured in the hypoxic condition using flow cytometry. The number in each quadrant represents the percentage of the gated live cells. D and E, Expression of vascular growth factor and endothelial cell marker genes in the pCSCs responding to hypoxia. The cells were harvested at day 1, 2, and 3 of cultures and analyzed by RT-PCR (D), or harvested at day 4 and analyzed by real-time PCR (E). Data shown are representatives from 3∼4 experiments, and the data of D was also quantitated and shown in Fig. S3.

Since hypoxia can induce progenitors to differentiate into endothelial cells [39], we examined whether pCSCs could differentiate into endothelial-like cells in responding to hypoxia. 2C4 cells were cultured in a hypoxic culture system to induce their differentiation (Fig. 3). Four days after cultivation in the matrigel-containing or suspension medium, pCSCs were altered significantly in morphology under hypoxic condition compared to normoxic culture condition (Fig. 3a). Elongated endothelial-like cells were observed, especially in the suspension cultures (Fig. 3A), despite no typical endothelial cell tube was formed. Some elongated cells were aligned in tandem (data not shown). Under normoxic condition, pCSCs retained round in morphology (Fig. 3A). In addition, hypoxic culture condition also significantly suppressed pCSC expansion i*n vitro* (Fig. 3B). This may be in part related to differentiation-induced cell death [15], because apoptotic cells were found in the colonies of differentiating cells (Fig. 3A, arrows). Flow cytometric analysis demonstrated that the frequency of CD45^+^CD31^+^ endothelial precursors was significantly increased in the hypoxic cultures compared to that in normoxic cultures [40], despite overall frequency was lower than we expected (Fig. 3C). The low frequency of CD31^+^ cells might reflect that only small numbers of pCSCs could differentiate into endothelial-like cells. This may be related to their genetic instability and environmental cues provided by the culture system. Note that the level of CD31 expression was proportional to that of CD45 expression (Fig. 3C), and the isotype antibody control excluded the possibility of non-specific staining. Some pCSCs cultured in normoxic condition also expressed CD31 (Fig. 3c), probably correlating with constitutive expression of CD31 mRNA in pCSCs (Fig. 3D & Fig. S3).

Interestingly, most pCSCs cultured in hypoxia condition remained CD31-negative (Fig. 3c), suggesting that pCSC-derived endothelial-like cells were essentially abnormal. Thus, we further examined the effect of hypoxia on the expression of angiogenic/vasculogenic genes as well as endothelial cell-related genes in pCSCs, including VEGF, Ang-1, and VEGFR-2, hypoxia-induced factor-1α (HIF-1α), CD31, and endoglin (CD106). Fig. 3D shows a representative experiment of RT-PCR analysis of kinetic expression of the genes in pCSCs under normoxic or hypoxic culture condition, and the results were quantitated as showing in Fig. S3. The genes examined were constitutively expressed in pCSCs cultured in normoxic condition (Fig. 3d & Fig. S3A). However, VEGF and HIF-1α were expressed in higher level than Ang-1, VEGFR-2, endoglin, and CD31 (Fig. S3A). Interestingly all the genes examined in pCSCs were unchanged or down-regulated within 48 hrs of hypoxic culture. At 72 hrs of culture, hypoxia induced an increased expression of Ang-1, VEGFR-2, endoglin and CD31, but not HIF-1α and VEGF (Fig. S3B). In particular, Ang-1 was dramatically up-regulated by ∼5 folds (Fig. S3B). The expression pattern was also detected by real-time PCR at 96 hrs of culture (Fig. 3E, compared Fig. S3A). The transcripts of Ang-1, VEGFR-2, endoglin and CD31 were significantly up-regulated (>2 folds increase); whereas VEGF and HIF-1α remained unchanged (<1 fold reduction). The unsignificant alteration of VEGF and HIF-1α in hypoxic culture condition might be related to their high level of constitutive expression in pCSCs (Fig. S3B) or the abnormality of pCSCs as endothelial progenitors. The results suggest that pCSCs, like *in vivo* ([15] & Fig. 1), can also differentiate into endothelial-like cells in responding to hypoxia *in vitro*.

### Precancerous stem cells are more potent than TMCs in vasculogenesis

**Figure 4:**
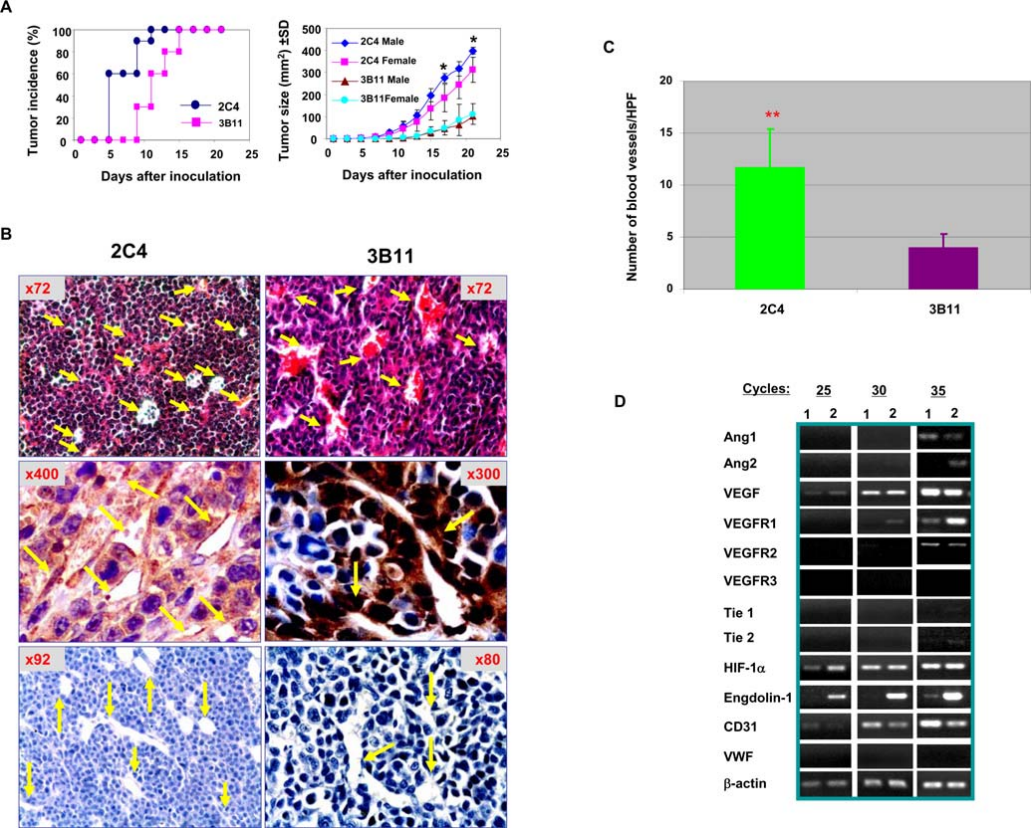
Comparison of vasculogenic capacity between pCSCs and TMCs. SCID mice were inoculated s.c. with pCSCs (2C4) at left groin and TMCs (3B11) at right groin (5×10^6^/mouse). Tumor incidence and size were monitored every other day (A), and pCSC- or TMC-derived blood vessels were analyzed by H & E and IHC staining of paraffin-embedded tumor sections (B), and the blood vessels in each section were counted under high power microscopy (C). Constitutive expression of angiogenesis-related genes between pCSCs and TMCs was compared before inoculation. A, tumor incidence and size: *, p<0.05; when compared between male (n = 5) and female (n = 5) mice. B, Analysis of pCSC- or TMC-derived tumor vasculature: upper panel: H. & E staining; middle panel: IHC staining specific for neomycin; and bottom panel: IHC staining controls with normal rabbit IgG as primary antibody. Arrows in the middle and bottom panels indicate neomycin-positive or negative TVECs or blood vessels at various developing stages. C, Comparison of the numbers of neomycin^+^ blood vessels between pCSC- and TMC-derived tumors: Neomycin^+^ blood vessels were counted under the 400× field of light microscope, and expressed as the number of per high-power field (HPF). Each tumor was counted for three successive sections, and 5 tumors were counted per group (**, p<0.01, as compared between pCSC and TMC-derived tumors). D, Constitutive expression of angiogenesis-related genes between pCSCs and TMCs: The 2C4 and 3B11 cells were harvested at log-phase of growth and analyzed by semiquantitative RT-PCR for 25, 30 and 35 cycles, respectively (Lane 1, 2C4; lane 2, 3B11).

The capacity of vasculogenesis may determine the rate of tumor growth. We have reported that pCSCs are more potent in tumorigenesis in SCID mice than differentiated TMCs from the same lymphoma [15], [23]. Thus, we compared the vasculogenic capacity between pCSCs and TMCs. pCSCs (2C4) and TMCs (clone 3B11) were transplanted s.c. into SCID mice, respectively. Consistently with our previous report [15], pCSCs had more potent capability than differentiated TMCs to form a new tumor. The tumors in the mice that received 2C4 cells were palpable at least 5 days earlier than those in the mice that were injected with 3B11 cells (Fig. 4a; top panel). Moreover, the growth kinetics of pCSC-derived tumors was much faster than TMC-derived tumors, and the size of pCSC-derived tumors was 4 ∼5 times bigger than that of TMC-derived tumors at the time of harvest (Fig 4a, bottom panel). Interestingly, the sex hormones appeared to affect the growth of pCSC-derived but not TMC-derived tumors, because the former but not the latter grew significantly faster in male mice than in female mice (Fig. 4a), suggesting that estrogen affect pCSC but not TMC expansion *in vivo*.

The different growth rate between pCSC- and TMC-derived tumors may be associated with their capacity of tumor vasculogenesis. Histological analysis revealed that the vasculature in pCSC-derived tumors was much more mature and dense than in TMC-derived tumors (Fig. 4b). Most capillary cells exhibited endothelial-like morphology in pCSC-derived tumors, while most cells lining up the vessel wall of TMC-derived tumors were monocytic in morphology (Fig. 4b). Most vessels in the TMC-derived tumors were at early stage of formation, i.e., tubular sinus formation, and some of which were unexpectedly filled with red blood cells [Fig 4b (3B11); top panel]. To determine the cell origin of tumor vasculature, we examined neomycin-expressing cells within tumor vasculatures by immunohistochemical (IHC) staining, as both 2C4 and 3B11 cells carried neomycin gene integrated into genome [15]. As shown in Fig. 4b (middle panel), most cells lining on vascular lumens were derived from pCSCs or TMCs, because they were strongly stained by mAb to neomycin. Note that the expression pattern of neomycin was different between pCSCs and TMC-derived tumors. In pCSC-derived tumors, neomycin was strongly detected in cytoplasm but weakly in nuclei; however, it was strongly detected in both cytoplasm and nuclei in TMC-derived tumors. The number of neomycin-positive blood vessels per high-power field (HPF) was significantly higher in pCSC-derived tumors than in TMC-derived tumors (Fig. 4c). The results suggest that both pCSCs and TMCs have the potential of vasculogenesis, although the vasculogenic capacity between them is different. Thus, the difference of growth rate between pCSCs and TMCs appears to be correlated with their capacity of vasculogenesis.

To determine whether the differential capacity of vasculogenesis between the pCSCs and TMCs was associated with differential activation of vasculogenic genes between them, we examined constitutive expression of vasculogenic and endothelium-related genes in these lines using semiquantitative RT-PCR, including VEGFR1/2/3, VEGF, Ang-1/2, Tie1/2, HIF-1α, endoglin, CD31 and von Willebrand factor (VWF). While pCSCs and TMCs expressed comparable levels of HIF-1α, VEGFR-2, VEGF and Ang-1 transcripts, the pCSCs expressed lower levels of transcripts of VEGR1 and Tie1/2 than TMCs, and even did not constitutively express Tie1, Tie2, and Ang-2 (Fig. 4d). Transcripts of endothelial cell-related genes CD31 and endoglin (CD105), but not VWF were detected in both pCSCs and TMCs (Fig. 4d). Taken together, the difference in tumor vasculogenic capacity between pCSCs and TMCs seems not associated with the expression levels of vasculogenic genes, but may be related to the stem-like property of pCSCs [15].

### Human tumor cell-derived endothelial-like cells are abnormal in phenotype and function

To determine whether human tumor vasculature was also derived from tumor vasculogenic stem/progenitor cells (TVPCs) [16], we examined the origin of tumor vasculatures in human tumor xenografts. Since there is no human pCSC clone available, we used human tumor cell lines, because they may contain pCSCs and/or CSCs [16], [24]. Human leukemic cell line MV411 [41], breast cancer cell lines MDA-MB-231 (ATCC HTB26) or MDA-MB-468 (ATCC HTB132) were injected s.c. into SCID CB17 mice, all of them grew out in the recipients (data not shown). The tumors were removed at the size 10∼15 mm in diameter, and subjected to histological and IHC analysis, as described above. The tumor cell-derived vasculogenic progenitors or vascular endothelial-like cells, which were lining on vessel walls, were determined by anti-human CD45, CD31, CD34, or VWF.

**Figure 5:**
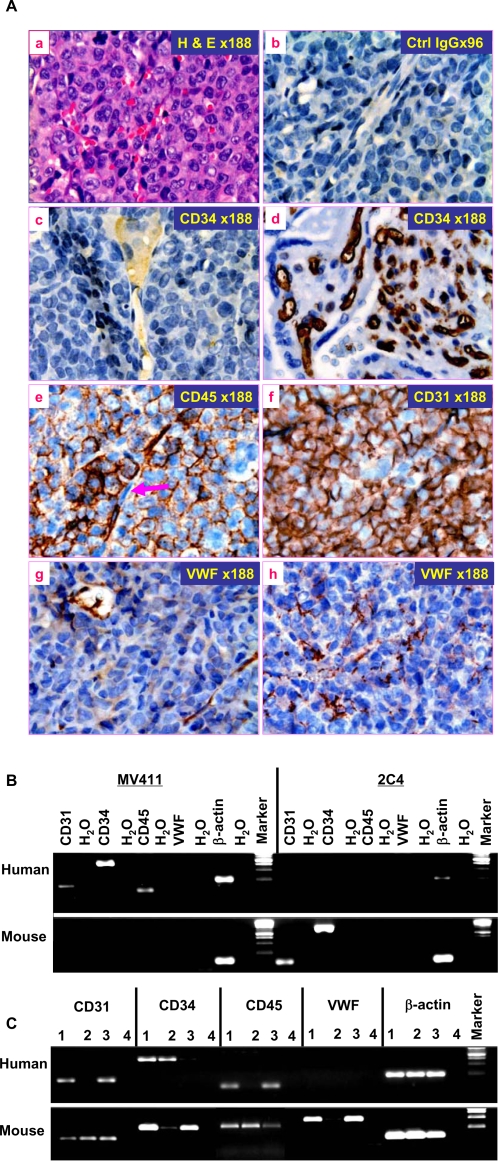
Vasculogenic capacity of human tumor cell lines. Human Leukemia/lymphoma cell line MV411 was injected s.c. into groin of SCID mice (5 ×10^6^/mouse; n = 4). MV411 developed into solid tumor palpable at day 15 of injection and the tumors were harvested 26 days after injection. The tumors were harvested for RNA extraction or fixed in 10% formaldehyde. A, Activity of TVPCs in human tumor cell lines: the sections of xenograft tumors were stained with H. & E. (a) or stained immunohistochemically with rabbit IgG (b) or rabbit anti-human CD34 (c & d), CD45 (e), CD31 (f) or VWF (g & h), followed by HRP-conjugated secondary antibody. In the d, the sections of human placenta were stained as positive control for CD34 in the same slides of c. The arrow in e indicates a CD45-negative endothelial-like cells lining on the wall of a blood vessel. Original magnification of micrographs: ×400 (numbers in the micrographs indicate real magnification shown). B & C, Expression of endothelial-related genes in the human MV411 tumor cells before (B) and after transplantation (C): Before transplantation, the MV411 and 2C4 cells were harvested at log-phase of growth, and extracted for total RNA. The mRNAs from MV411 and 2C4 cells were probed with both human and murine primers specific for CD45, CD34, CD31, VWF and β-actin mRNAs, respectively. Note that murine primers of β-actin cross-reacted to human β-actin, vice versa. The data shown are representative of three experiments. Murine 2C4 cells were used as species specific negative and positive controls for human- and murine specific primers, respectively (B). After transplantation, three MV411 cell-derived tumors were extracted for total RNA as described in A and probed with human and murine primers of CD45, CD34, CD31, VWF and β-actin, respectively. Lane 1∼3: each individual tumor; Lane 4: H_2_O, used for technical control for RT-PCR (C).

In the MV411-derived xenograft lymphomas, a high density of capillary network was observed, and some capillaries were filled with RBCs (Fig. 5A-a). Most blood vessels were derived from MV411 cells because they were strongly stained by anti-human CD45 and CD31 (Fig. 5A-e, f). Some mosaic vessels were observed, in which few endothelial-like cells did not express CD45 (Fig. 5A-e, arrow), suggesting that they might be derived from host endothelial progenitors. However, it was not certain whether these CD45-negative endothelial-like cells were derived from the progenitors of recipients, because tumor vascular endothelial cells could be abnormal and thus may lack CD45 [42]. Interestingly CD 34 in all tumor vascular endothelial cells was undetectable (Fig. 5A-c) and VWF expression was remarkably variable among tumor vascular endothelial-like cells (Fig. 5A-g, h). The results suggest that tumor cell-derived vascular endothelial-like cells were defective in phenotype and functions. The failure to demonstrate CD34 in tumor vascular endothelial cells was not caused by the quality of anti-CD34 antibody because the same antibody strongly stained the normal endothelial cells in the placental sections mounted on the same slides (Fig. 5A-d). To further verify that human vascular endothelial markers detected by IHC staining were truly derived from MV411 cells, we further examined the transcripts of CD31, CD34, CD45 and VWF in MV411 cells before (Fig. 5B) and after transplantation (Fig. 5C). MV411 cells constitutively expressed the transcripts of CD31, CD34, CD45 but not VWF, which were specifically detected by respective human-specific but not murine-specific primers (Fig. 5B). The transcripts of these markers were also detected in MV411 cell-derived tumors although with variable levels among individuals (Fig. 5C). The transcripts of murine CD31, CD34, CD45 and VWF were also detected in some tumors with variable levels (Fig. 5C), suggesting that host endothelial progenitors were involved in tumor angiogenesis to some extent. The specificity of these primers was verified by their respective capability to detect corresponding mRNAs in MV411 and 2C4 cell lines (Fig. 5B). Since 2C4 did not express CD45 [15], murine CD45 mRNA was not detected in 2C4 cells (Fig. 5B), but was detected in xenograft tumors (Fig. 5C). Overall, the results strengthen the conclusion that MV411 cells contain TVPCs.

Consistently, in the tumor xenografts derived from human breast cancer cell lines (MDA-MB-231 and MDA-MB-486), tumor cell-derived endothelial-like cells were observed. However, little CD45^+^, CD31^+^, CD34^+^, and VWF^+^ cells were detected among tumor endothelial-like cells. This is unlikely associated with angiogenesis, because recipient-derived vascular endothelial cells were not detectable in most capillary network using anti-murine CD31 antibody (not shown). Again, the abnormal phenotype of tumor vascular endothelial-like cells may be related to the defective differentiation of the TVPCs derived from tumor cell lines.

**Figure 6:**
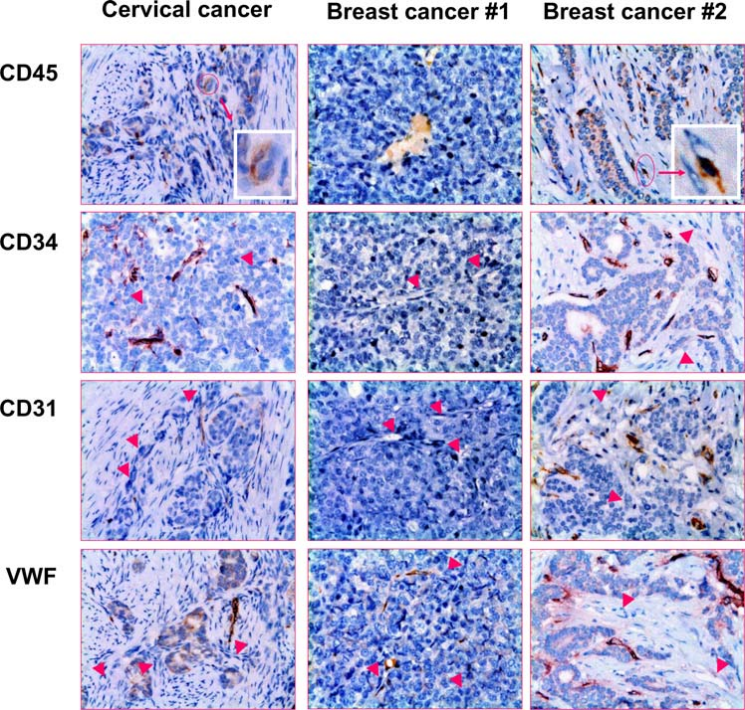
Defective phenotype and function of human TVECs. The sections of human cervical (n = 25) and breast cancer (n = 5) specimens were stained immunohistochemically with mAbs to CD45, CD31, CD34, or VWF. None of the cases examined demonstrated normal profile of endothelial cell markers, and none of the markers examined was detected in all tumor blood vessels. The insets indicate CD45^+^ endothelial-like cells within blood vessels; and arrow heads indicate that the endothelial-like cells lining upon blood vessels did not express relevant markers examined. The data shown are representative micrographs of human cervical and breast cancer. Original magnification: ×400; final magnification shown: ×72.

To further confirm that TVECs were defective in phenotype and function, we examined expression of CD45, CD34, CD31 and VWF in the native human cervical and breast cancers. As expected, the phenotype and function (VWF expression) of tumor endothelial cells in these cancers were essentially abnormal (Fig. 6). The expression pattern of CD45, CD34, CD31 and VWF in both cervical and breast cancer was highly variable between individuals being examined (Fig. 6). No consistent phenotypic pattern between individuals or between types of cancer could be drawn based on IHC staining. Few CD45^+^ vascular cells might represent TVPCs (Fig. 6). Overall the results suggest that most, if not all, human TVECs are derived from TVPCs, and thus are defective in phenotype and function.

## Discussion

Tumor vascularization is critical for tumor growth, invasion and metastasis. Traditionally tumor vasculature has been considered to be derived from pre-existing blood vessels through a process of angiogenesis [1]–[3], [43]. However, increasing data have suggested that tumor vasculature may be derived from cancer cells through a process of vasculogenesis [12]–[14]. In this study, we for the first time provide evidences that tumor vasculature can be derived at least from pCSCs, a population of cancer cells representing premalignant stage of developing CSCs [15], [16], suggesting that pCSCs are important for tumor vasculogenesis. This finding not only establishes that pCSCs can serve as progenitors for tumor vasculogenesis (TVPCs) but also explain why current anti-angiogenic cancer therapy trials are facing serious challenge [25].

Although the prevailing concept argues that tumor neo-vasculature is derived from pre-existing blood vessels or bone-marrow (BM)-derived circulating endothelial progenitors [4], [9], it has been shown that the frequency of BM-derived endothelial cells were very low in tumor neo-vasculature [10], suggesting that tumor vasculature may be derived from tumor cells [12], [13], [44]. However, there is no evidence so far directly confirming that tumor cells can serve as a progenitor for tumor vasculogenesis, despite some studies have indirectly supported the hypothesis [12], [13], [44]. In this study, we directly demonstrate that pCSCs can serve as progenitors for tumor vasculogenesis. The conclusion is supported by the following evidences: first, the pCSCs have the potential to differentiate into endothelial-like cells *in vivo* [15] and *in vitro*; second, pCSC-derived TVECs were dominant in tumor vasculature; third, some pCSCs constitutively expressed the receptor VEGFR-2 (Flk-1) and ligand VEGF, which may drive pCSC-derived TVPCs to differentiate into endothelial cells probably through an autocrine mechanism [45]; fourth, instead of freezing, fixation of pCSC-derived tumors with formalin to preserve fine structure of tumor vasculatures allowed us to directly visualize the pCSC-derived GFP^+^ endothelial cells that aligned on vessel walls; fifth, the pCSCs responded well to both hypoxia and angiogenic cytokines; Sixth, the blood vessels in human cancer xenografts were also overwhelmingly derived from the transplanted human tumor cell lines, which should contain pCSCs and/or CSCs [22], [24]. Subcutaneous transplantation of human MV411 cells into SCID mice led to the formation of xenograft lymphoma, in which almost all vascular endothelial cells were derived from MV411 cells. Finally, TVECs in human native cancers such as cervical and breast cancers were found to be defective in phenotype and function (VWF expression).

It has long been observed that the endothelial cells of tumor vessels are abnormal concerning their morphology and phenotype as revealed by electronic microscopy [44], [46] and IHC staining [47], respectively. This abnormality exhibited as the mosaic blood vessels, in which, e.g., both CD31^+^CD105^+^ endothelial cells and CD31^−^CD105^−^ cells formed luminal surface [47]. The abnormal phenotypes of tumor vascular endothelial cells had been ascribed to the tumor cells that aligned on the defective walls of tumor vessels [47], or to the tumor environments which caused abnormal differentiation of normal endothelial progenitors [48]. However, our studies indicate that these abnormal vascular cells, at least some of them, may be derived from tumor cells such as pCSCs and CSCs rather than from normal progenitors. Expression of one or two endothelial markers on tumor vascular endothelial cells does not seem to sufficiently prove that they are derived from normal progenitors. Examination of vascular endothelial cell markers in tumor xenografts and the specimens of human cervical and breast cancer revealed that little normal endothelial cells could be detected in these cancers. None of the cases examined of each type of cancer expressed a complete array of normal endothelial markers, suggesting that these endothelial cell markers are randomly expressed depending on environmental cures. Consistently, pCSCs in responding to hypoxia expressed little up-regulated CD31, although their morphology was changed into endothelial-like and their genes for vascular growth were up-regulated. The pCSC-derivation of TVECs may explain why they are phenotypically multifaceted [42]. It is likely that most tumor vasculature are derived from pCSCs and/or CSCs, especially from those having the potential to differentiate into endothelial-like cells [15], [16]. The markers for TVPCs remain to be elucidated. However, VEGFR-2 might be a candidate marker of TVPCs, because VEGFR-2-mediated signaling is required for both angiogenesis and vasculogenesis [6], [8], [45], [49], [50].

**Figure 7:**
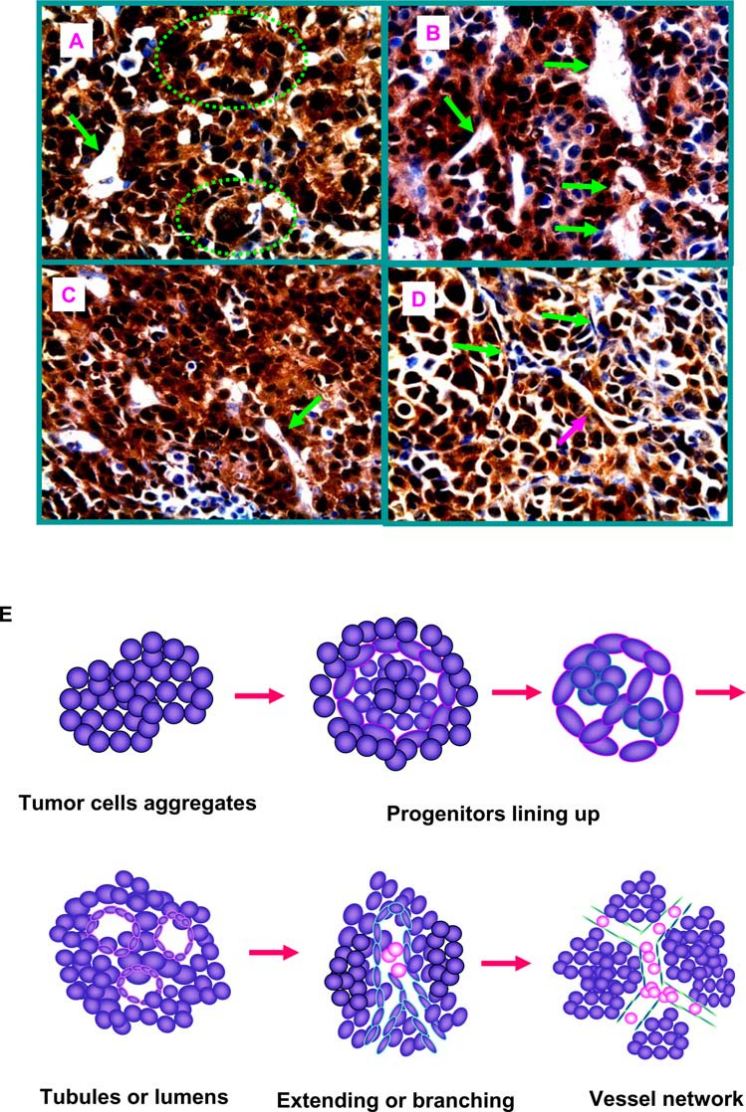
The putative cellular mechanism for tumor vasculogenesis. TVPCs within tumor cell aggregates line-up along the branching lumen (A, circles), and form tubes (A & B, green arrows). The tubes are extended, elongated, and become neo-vasculatures (C, green arrow). The newly formed vasculature (neomycin^+^; red arrow) merged with host blood vessels (neomycin^−^; green arrow) to form the neo-vasculature networks (D). E, A schematic process of tumor vasculogenesis illustrated based on A∼D. The figure A is derived from a 3B11 tumor. Original magnification of micrographs: ×600.

A critical question, however, remains to be answered: what is the status of angiogenesis in tumor neo-vascularization? While Lyden et al showed that impaired recruitment of BM-derived endothelial and hematopoietic precursor cells blocked tumor angiogenesis and growth [9], Larrivee et al demonstrated that the contribution of BM-derived precursor to tumor angiogenesis is minimal [10]. The argument appears to be reconciled by the recent finding showing that BM-derived precursors seems dominant in early tumor but greatly diluted in later tumor [51]. Combined with our findings, we propose that angiogenesis is required for efficient tumor vasculogenesis. In early tumor, endothelial cells from pre-existing blood vessels or circulating endothelial progenitors may proliferate and sprout surrounding cancer cell aggregates; simultaneously tumor cell-derived progenitors start a process of vasculogenesis. In animal models as well as human tumor xenograft models, we observed various stages of developing blood vessels, as outlined in Fig. 7. Initially the progenies of pCSCs or CSCs form an aggregate in tumorigenic niches (Fig. 7a, e). With the enlargement of the aggregates, pCSCs or CSCs that have the properties of TVPCs line up to form branching lumens and tubes (Fig. 7b, e). The tubes were extended and elongated (Fig. 7c, e), and the newly formed vasculature merged with host vessel sprouts from pre-existing blood vessels surrounding tumor capsule to form functional neo-capillary network (Fig. 7d, e). Without angiogenesis, tumor vasculogenesis might be aborted [9]. This model emphasizes that the formation of tumor vasculature requires the mechanisms of both angiogenesis and vasculogenesis, and may explain the respective roles of angiogenesis and vasculogenesis in tumorigenesis (Fig. 7e).

Another important issue is the factors that affect tumor angiogenesis and vasculogenesis. It is well-known that both angiogenesis and vasculogenesis are mediated by the same growth factors and receptors. Most important factors are VEGF and Ang-1/2, which bind receptors VEGFR-1/2 and Tie1/2, respectively [52], [53]. Among them, VEGFR-2-mediated signaling is critical for vasculogenesis in embryonic and tissue vasculogenesis [8], [54], and Ang-1/2 and Tie-1/2 are mainly responsible for angiogenesis [53], [55]. While the vascular growth factors mainly determine the capacity of tumor angiogenesis [1], [22], their effect on tumor vasculogenesis seems to be limited. The notion is supported by the fact that TMCs that produced comparable or even higher levels of growth factors or receptors had much less capacity of tumor vasculogenesis, when compared to pCSCs. The different vasculogenic capacity between pCSCs and TMCs does not seem to associate with expression of VEGFR-2 on these cells, because they expressed comparable VEGFR-2 transcripts. TMCs expressed higher levels of VEGFR1 and Tie2 than that of pCSCs. VEGFR1 has been shown to limit the mitogenic function of VEGFR-2 [56], [57] and, thus might limit VEGF-induced vasculogenesis. Tie2/Ang-2 signaling has been shown anti-angiogenic [58], [59]. Whether vasculogenic capacity of TMCs is limited by these two factors remains elusive.

As discussed above, examination of human cervical and breast cancer specimen revealed that most blood vessels in invasive cancer were derived from tumor cells because little normal endothelial cells were detected. This observation suggests that in addition to capability to produce vascular growth factors, the status of vasculogenesis in tumor may be determined by frequency and intrinsic properties of tumor cell-derived vascular progenitors. This explains why most clinical trials of anti-angiogenic therapy have failed to block tumor growth [25]. Currently anti-angiogenic drugs are screened by using *in vitro* angiogenic models composed of normal endothelial progenitors [25]. As a result, while screened drugs are effective on normal endothelial progenitors, they may be resisted by TVPCs. Thus, pCSCs and CSCs may be main targets for anti-angiogenic cancer therapy. In addition, cytokines such as GM-CSF, FL and IL-13 can up-regulate VEGFR-2 expression on pCSCs. Whether these factors promote pCSC-mediated vasculogenesis needs further investigation.

In addition to pCSCs, we also show that TMCs have the potential of tumor vasculogenesis, suggesting that pCSCs are not a sole population having activity of TVPCs. This is not surprising, because monocytes are highly plastic in differentiation [23]. It has been reported that monocytes or myeloid cells have the capability to transdifferentiate into endothelial cells [60]–[62]. Monocytes have been shown to play an important role in tumor angiogenesis [63]. On the other hand, activated monocytes can secrete soluble (s) VEGFR1 to inhibit angiogenesis [64]. It should be noted that vasculogenic capacity of TMCs was less than that of pCSCs, despite both of them are derived from the same tumor [15], [23], and that TMCs expressed high level of VEGFR1. Whether TMCs can produce sVEGFR1, leading to defective tumor vasculogenesis; or their vasculogenic capacity is hierarchically reduced when they developed from pCSCs. Further elucidating these issues will facilitate understanding of the mechanisms underlying tumor vasculogenesis.

A solid tumor has been considered a neo-organ in the body, consisting of various components including stromal and parenchymal cells; the latter are composed of pCSCs, CSCs and terminal differentiated cancer cells [16]. Its malignancy is determined essentially by the degree of uncontrollable cell growth, neo-vascularization, and the capacity of metastasis. Resembling normal organs, which may be replenished by tissue stem cells, tumor may be replenished by pCSCs and/or CSCs. On the basis of our previous finding [15], we here further reveal that pCSCs can serve as progenitors for tumor vasculogenesis (TVPCs) as well as progenitors of RBCs in tumors.

## Methods

### Mice, cell lines, and reagents

SCID CB17 mice were used at age of 8–12 wk. We bred and maintained the mice in an animal pathogen-free facility at The Ohio State University Medical Center. Murine pCSC clones 2C4 and 2C4G2 and monocytic tumor cell (TMC) line 3B11 from the same tumor were generated and maintained in our laboratory [15], [23]. The GFP-expressing 2C4G2 cells were derived from 2C4 clone [15]. 2C4 and 3B11 carry neomycin gene in the genome [15]. Human leukemic cell line MV411, and breast cancer cell lines MDA-MB-231 and MDA-MB-486 were maintained in our laboratory. Murine mAbs to human CD31, CD45, and VWF (von Willebrand factor VIII) as well as isotype mAbs were purchased from Dako (Denmark); and murine mAb to human CD34 mAb was purchased from Immunotech Inc. Monoclonal rabbit anti-GFP antibody was purchased from Epitomic Inc (Burlingame, CA). Polyclonal rabbit anti-neomycin antibody was provided by Upstate Cell Signaling Solutions (Lake Placid, NY). Recombinant murine GM-CSF, Flt3 ligand (FL), IL-3, IL-4, IL-6, IL-7 and IL-13 were purchased from PeproTech. Fluorescent dye-conjugated antibodies were purchased from BD Science.

### Cell culture

The cell lines were maintained in R10F (RPMI 1640 plus 10% fetal calf serum supplemented with 5 mM glutamine, 50 µM 2-mecaptoethonal, 100 U/ml penicillin, and 100 µg/ml streptomycin) [15], [23]. To investigate the effect of cytokines on VEGFR-2 expression in pCSCs, the cells were cultured in R10F for 3 days in the absence or presence of cytokines alone or in combination, including recombinant murine GM-CSF, Flt3 ligand (FL), IL-3, IL-4, IL-6, IL-7 and IL-13. To induce pCSC differentiation into endothelial cell-like cells in vitro, the pCSCs were cultured in hypoxic medium as indicated in figure legend.

### Tumor transplantation

SCID CB17 mice were injected s.c. with 5×10^6^ 2C4, 2C4G2, 3B11, MDA-MB-231, MDA-MB-486, or MV411 cells. Tumor incidence and size were monitored starting from 1 wk after inoculation, once every other day. The mice were sacrificed when one of the mice in a group bearing tumor about 10∼15 mm in diameter. Tumors were harvested for histological and IHC staining [15].

### RT-PCR

Total RNA was extracted from cell lines. The cDNA was generated by reverse transcription using SuperScript III First-Strand Synthesis System ( Invitrogen, CA) and oligo (dT) in a 20 µl reaction containing 1 µg of total RNA, which was pretreated with RNase-free DNase I (Qiagen, CA) to eliminate contaminating genomic DNA. Briefly, an aliquot of 0.5 µl cDNA was used in each 25 µl PCR reaction, using Platinum® Taq DNA Polymerase High fidelity (Invitrogen, CA). The following conditions were used: an initial denaturation at 95°C for 5 min followed by denaturation at 94°C for 30 seconds, annealing at 58°C for 30 seconds and extension at 68°C for 1 min for a total of 25, 30 or 35 cycles. PCR products were analyzed by 2.0% agarose gel. The sequence of the primers used is listed in supplementary Table S1.

### Real-time PCR

Real-time PCR was performed on ABI 7500 Real-Time PCR System (Applied Biosystems, Inc., CA). cDNA was combined with primer sets and Power SYBR® Green PCR Master Mix (Applied Biosystems, Inc., CA). The sequence of the primers used is listed in supplementary Table S1. The PCR conditions were as follows: 95°C for 10 s, 60°C for 1 min, 45 cycles. Gene expression levels were calculated relative to the house-keeping gene β-actin by using Sequence Detection Software (version1.3.1) (Applied Biosystems, Inc., CA).

### Induction of pCSC differentiation in hypoxic culture system

The pCSCs (5×10^4/^well) were cultured in 0.5 ml (hypoxia) and 2.0 ml (control) R10F medium with or without BD Matrigel™ (1∶4 dilution) for 4 days in 24-well plates in triplicate. The morphologic alteration was monitored under phase contrast microscope every day. In some experiments, the cells were harvested on day 1, 2, and 3 for RT-PCR or real-time PCR analysis of the genes of angiogenic factors and endothelial cell markers. For flow cytometric analysis, the cells were harvested and stained where indicated.

### Flow cytometry

Single cells harvested from cultures were stained with corresponding mAbs, and then analyzed by flow cytometry, as described previously [15], [23].

### Histological and immunohistochemical analysis

Tumor specimens were fixed in formalin and embedded in paraffin for pathological and immunohistochemical analysis, as described [15], [23]. Human specimens of cervical and breast cancer were provided by the Tissue Procurement Shared Resource (TPSR), Comprehensive Cancer Center, Ohio State University. Sections (4∼5 µm) were stained by H. & E. for pathological analysis, or immunostained with a monoclonal or polyclonal primary antibody followed by a horseradish peroxidase (HRP)-conjugated secondary antibody. The immunostained sections were counterstained with haematoxylin.

### Statistical analysis

Data were statistically analyzed by one-way ANOVA or student-T test. Two-tailed T test was performed except for where indicated. P-value≤0.05 was considered significant; and p-value≤0.01 was considered highly significant.

## Supporting Information

Figure S1 Multipotency of pCSCs in tumorigenesis 2C4G2 and 2C4 cell-derived tumor tissue sections were prepared and stained with rabbit mAb to GFP followed by HRP-conjugated goat anti-rabbit IgG, as described in Fig. 1. In the 2C4 cell-derived tumor sections, none of sections were GFP-positive (A & B), in striking contrast to the 2C4G2 cell-derived tumor sections (C ∼ H). Note that rabbit mAb to GFP is highly specific. The inset in A was enlarged as B; and the inset in C was enlarged as D, showing GFP-positive RBCs. The pCSCs-derived RBCs appeared to be smaller in size than host-derived GFP-negative RBCs (D, short green arrows). The level of GFP expression in pCSC-derived TVECs (C ∼ H), RBCs (F & G: arrows indicate RBCs expressing little or no GFP), and cancer cells (E & H) is variable.
(13.38 MB TIF)

Figure S2 The effect of angiogenic cytokines on pCSC expansion in vitro The pCSCs (2C4 clone) were cultured for 3 days in 2.0 ml of R10F (1×10^5^ cells/well) in 24-well plates supplemented with cytokine IL-3 (50 ng/ml), IL-4 (20 ng/ml), IL-6 (50 ng/ml); IL-7 (100 ng/ml), IL-13 (50 ng/ml), GM-CSF (40 ng/ml), or FL (200 ng/ml) alone or in combination (GM-CSF+IL-4; FL+IL-13; or IL-3+IL-6), as described in Fig. 2. Control cultures were absent from exogenous cytokines. The cells were harvested and trypan blue-excluded viable cells were counted. The data shown are from 3 independent experiments, and the p values were derived from one-tailed Student-T test when compared to control cultures.
(0.22 MB TIF)

Figure S3 Quantitation of angiogenic gene expression of pCSCs responding to hypoxia The results of Fig. 3D was quantitated using software ImageJ (1.37V, NIH). β-actin transcripts were used as internal control to normalize angiogenic gene expression. A, Constitutive expression of angiogenic genes in pCSCs (2C4) 24 hrs after cell splitting. B, Kinetics of angiogenic gene expression in pCSCs (2C4) responding to hypoxia.
(0.29 MB TIF)

Table S1 (0.09 MB DOC)
