# Supplementary figures and images for: Precancerous Stem Cells Can Serve As Tumor Vasculogenic Progenitors
Source: PLoS One. 2008 Feb 20;3(2):e1652. doi: 10.1371/journal.pone.0001652 (PMC2242848; doi:10.1371/journal.pone.0001652)

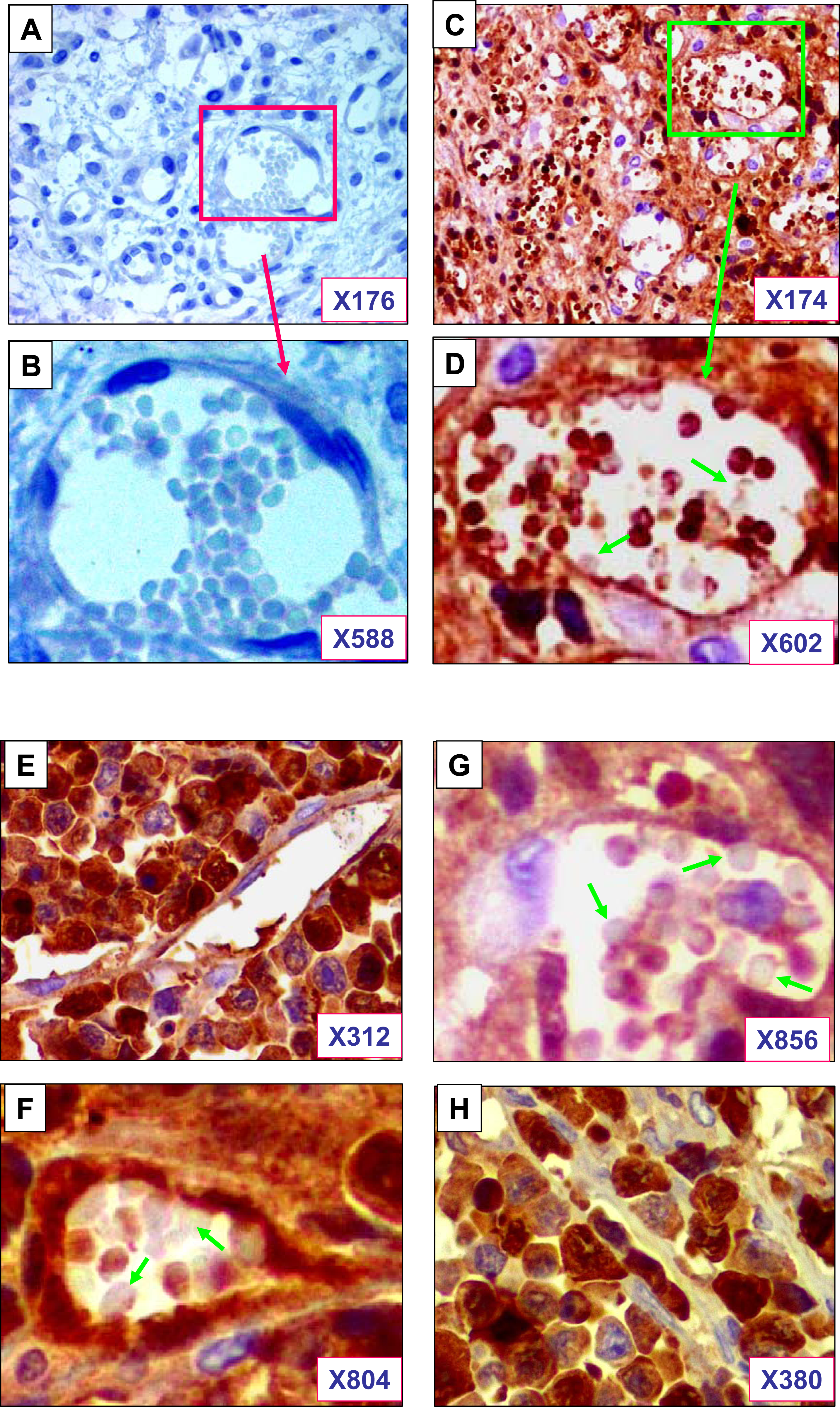

Supplement: Figure S1 — Multipotency of pCSCs in tumorigenesis 2C4G2 and 2C4 cell-derived tumor tissue sections were prepared and stained with rabbit mAb to GFP followed by HRP-conjugated goat anti-rabbit IgG, as described in Fig. 1. In the 2C4 cell-derived tumor sections, none of sections were GFP-positive (A & B), in striking contrast to the 2C4G2 cell-derived tumor sections (C ∼ H). Note that rabbit mAb to GFP is highly specific. The inset in A was enlarged as B; and the inset in C was enlarged as D, showing GFP-positive RBCs. The pCSCs-derived RBCs appeared to be smaller in size than host-derived GFP-negative RBCs (D, short green arrows). The level of GFP expression in pCSC-derived TVECs (C ∼ H), RBCs (F & G: arrows indicate RBCs expressing little or no GFP), and cancer cells (E & H) is variable. (13.38 MB TIF) [file pone.0001652.s001.tif]

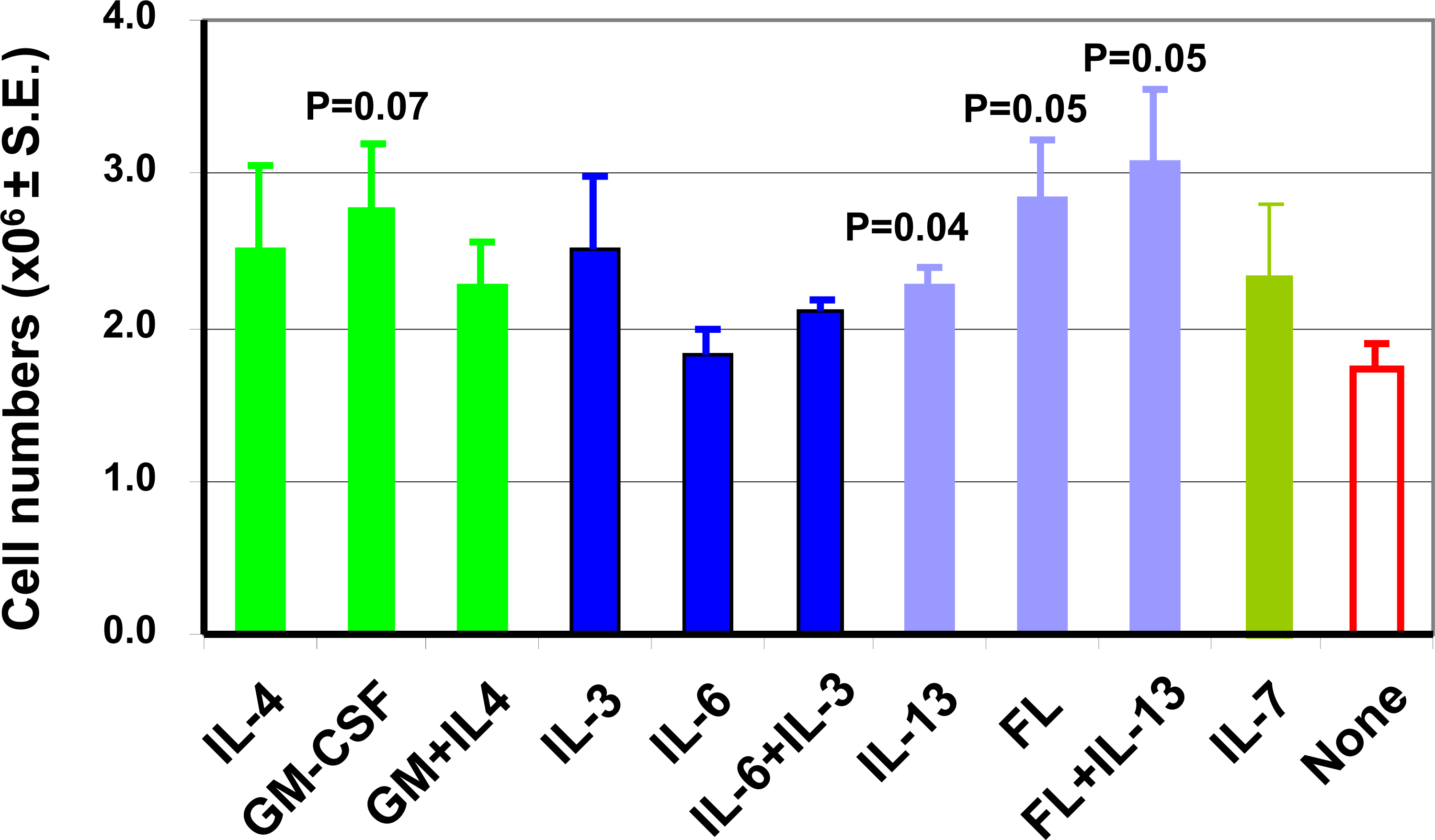

Supplement: Figure S2 — The effect of angiogenic cytokines on pCSC expansion in vitro The pCSCs (2C4 clone) were cultured for 3 days in 2.0 ml of R10F (1×10^5^ cells/well) in 24-well plates supplemented with cytokine IL-3 (50 ng/ml), IL-4 (20 ng/ml), IL-6 (50 ng/ml); IL-7 (100 ng/ml), IL-13 (50 ng/ml), GM-CSF (40 ng/ml), or FL (200 ng/ml) alone or in combination (GM-CSF+IL-4; FL+IL-13; or IL-3+IL-6), as described in Fig. 2. Control cultures were absent from exogenous cytokines. The cells were harvested and trypan blue-excluded viable cells were counted. The data shown are from 3 independent experiments, and the p values were derived from one-tailed Student-T test when compared to control cultures. (0.22 MB TIF) [file pone.0001652.s002.tif]

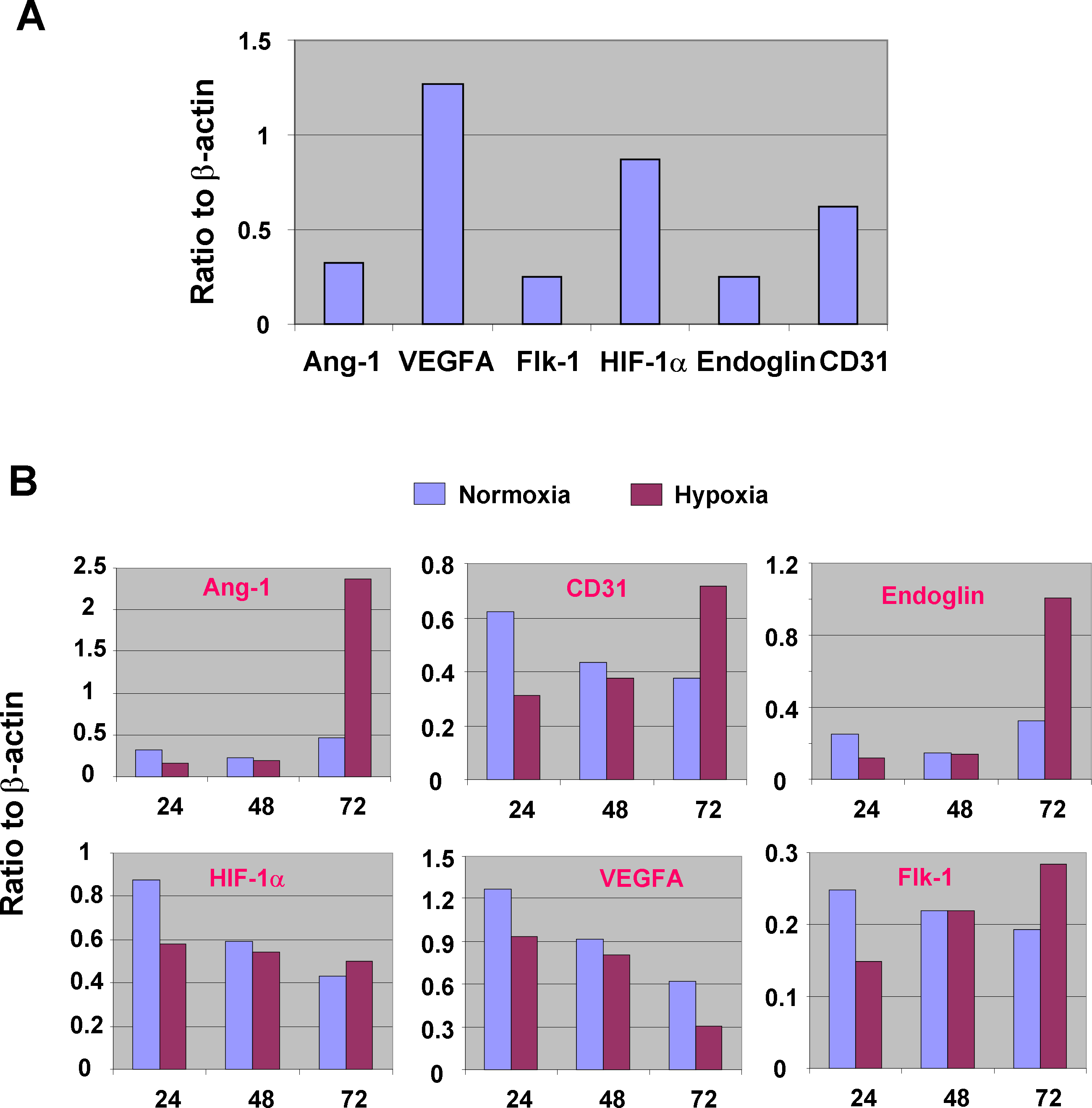

Supplement: Figure S3 — Quantitation of angiogenic gene expression of pCSCs responding to hypoxia The results of Fig. 3D was quantitated using software ImageJ (1.37V, NIH). β-actin transcripts were used as internal control to normalize angiogenic gene expression. A, Constitutive expression of angiogenic genes in pCSCs (2C4) 24 hrs after cell splitting. B, Kinetics of angiogenic gene expression in pCSCs (2C4) responding to hypoxia. (0.29 MB TIF) [file pone.0001652.s003.tif]
