# Supplementary material for: Precancerous Stem Cells Can Serve As Tumor Vasculogenic Progenitors
Source: PLoS One. 2008 Feb 20;3(2):e1652. doi: 10.1371/journal.pone.0001652 (PMC2242848; doi:10.1371/journal.pone.0001652)
Supplement: Table S1 — (0.09 MB DOC) [file pone.0001652.s004.doc]

**Supplementary Table 1. Primer sequence for RT-PCR and real-time PCR**

| **Murine primers:** | | | |
| --- | --- | --- | --- |
|  | | | |
| mANG1 FW | NM_009640 | 5'-CAT TCT TCG CTG CCA TTC TG-3' | 103bp |
| mANG1 RE |  | 5'-GCA CAT TGC CCA TGT TGA ATC-3' |  |
|  |  |  |  |
| mANG2 FW | NM_007426 | 5'-TTA GCA CAA AGG ATT CGG ACA AT-3' | 121bp |
| mANG2 RE |  | 5'-TTT TGT GGG TAG TAC TGT CCA TTC A-3' |  |
|  |  |  |  |
| mHIF-1a FW | NM_010431 | 5'-TGT GAA CCC ATT CCT CAT CCG TCA-3' | 134bp |
| mHIF-1a RE |  | 5'-TCC GGC TCA TAA CCC ATC AAC TCA-3' |  |
|  |  |  |  |
| mVEGF FW | NM_001025250 | 5'-GAG GAG ACT CTT CGA GGA GCA CTT-3' | 132bp |
| mVEGF RE | | 5'-GGC GAT TTA GCA GCA GAT ATA AGA A-3' |  |
|  |  |  |  |
| mTIE1 FW | NM_011587 | 5'-CAA GGT CAC ACA CAC GGT GAA-3' | 122bp |
| mTIE1 RE |  | 5'-GCC AGT CTA GGG TAT TGA AGT AGG A-3' |  |
|  |  |  |  |
| mTIE2 FW | NM_013690 | 5'-ATG TGG AAG TCG AGA GGC GAT-3' | 278bp |
| mTIE2 RE |  | 5'-CGA ATA GCC ATC CAC TAT TGT CC-3' |  |
|  |  |  |  |
| mMCD31 FW | NM_001032378 | 5'-GAG CCC AAT CAC GTT TCA GTT T-3' | 118bp |
| mMCD31 RE | | 5'-TCC TTC CTG CTT CTT GCT AGC T-3' |  |
|  |  |  |  |
| MCD34FW | NM_133654 | 5'-AGG CTC TGG AAC TCC ACA CAC TTT-3' | 450bp |
| MCD34RE |  | 5'-TTG GCC AAG ACC ATC AGC AAA CAC-3' |  |
|  |  |  |  |
| mVEGFR1 FW | NM_010228 | 5'-GAG GAG GAT GAG GGT GTC TAT AGG T-3' | 116bp |
| mVEGFR1 RE | | 5'-GTG ATC AGC TCC AGG TTT GAC TT-3' |  |
|  |  |  |  |
| mVEGFR2 FW | NM_010612 | 5'-GCC CTG CTG TGG TCT CAC TAC-3' | 114bp |
| mVEGFR2 RE | | 5'-CAA AGC ATT GCC CAT TCG AT-3' |  |
|  |  |  |  |
| mVEGFR3 FW | NM_008029 | 5'-TGG CAA ATG GTT ACT CCA TGA CCC-3' | 402bp |
| mVEGFR3 RE | | 5'-ACA TCG AGT CCT TCC TGT TGA CCA-3' |  |
|  |  |  |  |
| mENG FW | NM_007932 | 5'-ATC CAA CAC CAT CGA ACT AGG CCA-3' | 300bp |
| mENG RE |  | 5'-TAG ACT TCC TGG GAC AAG GTG CTA-3' |  |
|  |  |  |  |
| mvWF FW | NM_011708 | 5'-GCT TCC AAC TGA ACT GTG AGA CCT-3' | 509bp |
| mvWF RE |  | 5'-GGC TGT GAT GTC TTT GCA ATC AGG-3' |  |
|  |  |  |  |
| mACTB FW | NM_007393 | 5'-TGA ACC CTA AGG CCA ACC GTG AAA-3' | 136bp |
| mACTB RE |  | 5'-GAG TCC ATC ACA ATG CCT GTG GTA-3' |  |
|  |  |  |  |
|  |  |  |  |
| **Human primers**: | |  |  |
| hCD45 FW | NM_080922 | 5'-GGA AGT GCT GCA ATG TGT CAT TTC A-3’ | 113bp |
| hCD45 RE |  | 5'-GGA GGC CTA CAC TTG ACA TGC ATA CT-3’ |  |
|  |  |  |  |
| hCD31 FW | NM_000442 | 5'-AAC TGT GCC TGC AGT CTT CAC TCT -3' | 195 bp |
| hCD31 RE |  | 5'-TGC AGG GTC AGG TTC TTC CCA TTT-3' |  |
|  |  |  |  |
| hCD34 FW | NM_001773 | 5'-TCA ACA TGG CAA TGA GGC CAC AAC-3' | 235 bp |
| hCD34 RE |  | 5'-TGC AAG GCT AGT GCT AGT GGT TGA-3' |  |
|  |  |  |  |
| hvWF FW | NM_000552 | 5'-TCG TCA ACA CCT TTG ATG GGA GCA-3' | 219 bp |
| hvWF RE |  | 5'-AGG CAT AGG GCA TGG AGA CTC TTT-3' |  |
|  |  |  |  |
| hACTB FW | NM_001101 | 5'-CAT GTA CGT TGC TAT CCA GGC-3' | 250 bp |
| hACTB RE |  | 5'-CTC CTT AAT GTC ACG CAC GAT-3'. |  |

FW: forward; RE: reverse; ACTB: β-actin; VEGF: vascular endothelial growth factors; ANG1 or ANG2: Angiopoietin-1 or -2; HIF-1α: hypoxia-inducible factor 1, alpha subunit (basic helix-loop-helix transcription factor); VEGFA: vascular growth factor A; TIE1 or TIE2: tyrosine kinase with immunoglobulin-like and EGF-like domains 1 or 2; CD31: platelet/endothelial cell adhesion molecule (PECAM1); VEGFR1, 2 or 3: vascular endothelial growth factor receptor 1, 2 or 3; ENG: endoglin (CD105 antigen); VWF: von Willebrand factor.
